# DeepMPM: a mortality risk prediction model using longitudinal EHR data

**DOI:** 10.1186/s12859-022-04975-6

**Published:** 2022-10-14

**Authors:** Fan Yang, Jian Zhang, Wanyi Chen, Yongxuan Lai, Ying Wang, Quan Zou

**Affiliations:** 1 Shenzhen Research Institute of Xiamen University, Shenzhen, China; 2 Department of Automation, Xiamen University, Xiamen, China; 3 School of informatics/Shenzhen Research Institute, Xiamen University, Xiamen/Shenzhen, China; 4 Institute of Fundamental and Frontier Sciences, University of Electronic Science and Technology of China, Chengdu, China

**Keywords:** Deep learning, Electronic health records, Mortality risk prediction

## Abstract

**Background:**

Accurate precision approaches have far not been developed for modeling mortality risk in intensive care unit (ICU) patients. Conventional mortality risk prediction methods can hardly extract the information in longitudinal electronic medical records (EHRs) effectively, since they simply aggregate the heterogeneous variables in EHRs, ignoring the complex relationship and interactions between variables and the time dependence in longitudinal records. Recently deep learning approaches have been widely used in modeling longitudinal EHR data. However, most existing deep learning-based risk prediction approaches only use the information of a single disease, neglecting the interactions between multiple diseases and different conditions.

**Results:**

In this paper, we address this unmet need by leveraging disease and treatment information in EHRs to develop a mortality risk prediction model based on deep learning (DeepMPM). DeepMPM utilizes a two-level attention mechanism, i.e. visit-level and variable-level attention, to derive the representation of patient risk status from patient’s multiple longitudinal medical records. Benefiting from using EHR of patients with multiple diseases and different conditions, DeepMPM can achieve state-of-the-art performances in mortality risk prediction.

**Conclusions:**

Experiment results on MIMIC III database demonstrates that with the disease and treatment information DeepMPM can achieve a good performance in terms of Area Under ROC Curve (0.85). Moreover, DeepMPM can successfully model the complex interactions between diseases to achieve better representation learning of disease and treatment than other deep learning approaches, so as to improve the accuracy of mortality prediction. A case study also shows that DeepMPM offers the potential to provide users with insights into feature correlation in data as well as model behavior for each prediction.

## Background

Accurate identification of the mortality risk of patients plays an important role in assisting doctors in decision-making, improving diagnosis efficiency, rationally allocating medical resources and saving patients’ medical expenses. Conventional mortality risk prediction methods such as APACHE (Acute Physiology and Chronic Health Evaluation) [1] and SAPS (Simplified Acute Physiology Score) [2] usually utilize vital signs measurements of monitoring data, e.g., heart rate, systolic blood pressure and body temperature of patient, along with the demographic information to identify the mortality risk of ICU patients. Alternatively, machine learning approaches such as Support Vector Machine (SVM) [3] and Recurrent Neural Networks (RNN) [4] regard mortality risk prediction as a classification task, and usually provide more accurate prediction models. However, these models also rely on measurements of monitoring data or hand-crafted risk factors, while other rich information available in EHR, e.g., the diagnosis and prescription, are ignored, which always results in unsatisfactory performance in risk prediction.

Recently, the availability of EHR has demonstrated great potential in improving the performance of various kinds of medical applications including clinical risk prediction [5–8]. EHRs usually contain abundant patient information by recording various disease and treatment information, e.g., diagnoses, demographic information, laboratory tests and measurements and prescriptions of patients during their hospitalization, which provide a great opportunity to develop more accurate mortality risk prediction models. Traditional models are not suitable for EHR data analysis because they simply aggregate the heterogeneous variables in EHRs, ignoring the complex relationship and interactions between variables and the time dependence in longitudinal records. In this context, deep learning has been applied to capture the characteristics in heterogeneous EHR data, which makes up for the shortcomings of statistical and traditional machine learning methods.

### Traditional methods for mortality prediction

**Fig. 1:**
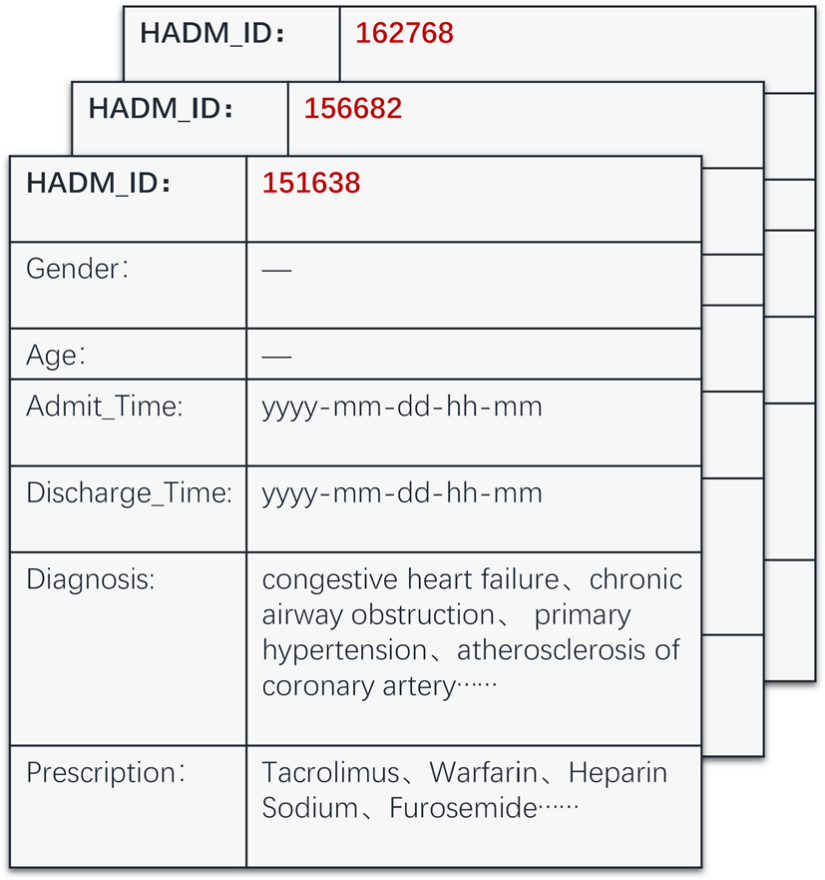
Three diagnostic records of a patient: HADM_ID refers to the record ID. Each record contains multiple variables, such as gender, age, admission time, discharge time, diagnosis codes, and prescription codes

In the 1980s and 1990s, researchers have constructed several professional scoring systems for the prognosis of ICU patients and describing the severity of disease and organ dysfunction, which have been widely used in clinical practice. Common scoring systems include APACHE [1], SAPS [2], MPM (Mortality Probability Model) [9] and their upgraded versions [10–12]. These scoring systems often intercept the vital signs monitoring data and demographic information (such as age and gender) of the patient in a certain window period (such as 24 h or 48 h) after the patient entering ICU as input, discretize the continuous variables, and finally output a risk score with a reference range. Since the beginning of this century, machine learning approaches have been used in mortality risk prediction, including logistic regression [13], support vector machine [3], decision tree [14], etc. These models also mostly use the short-term monitoring data of ICU patients, sometimes combined with APACHE or SAPS scores. However, the shallow structure of the above methods is difficult to fully utilize the potential information in EHR, which contains varying-length sequence with a long-term dependence as shown in Fig. 1. In short, the traditional risk-scoring tools are based on a small set of hand-crafted monitoring data or risk factors, while the traditional machine learning models such as SVM and LR also cannot well handle heterogeneous EHR data.

### Deep learning for EHR data analysis

Compared with traditional methods, deep learning can achieve complex function approximation through deep nonlinear network structure, and learn the internal relationship between data from varying-length longitudinal medical records.

#### Representation learning for EHR data

Representation learning of patient information is an important feature of EHR data research based on deep learning. It makes up for the defect that one-hot encoding [15, 16] cannot capture the semantic between features. Inspired by natural language processing, researchers applied skip-gram model [17–21] to EHR data to obtain high-quality distributed vector representation. In a recent study, Xiao et al. used the BERT-based deep learning model to obtain the Natural Language Representation of the EHR data to predict chronic cough patients [22]. With the development of GNNs in recent years, Rocheteau et al. proposed to combine GNN with LSTM to obtain relational information between similar patients in a graph [23]. Furthermore, to utilize the heterogeneity in EHRs, Liu et al. used preprocessing method to split the heterogenous EHR graph into multiple homogeneous graphs, and then used an end-to-end GNN to make prediction [24].

However, EHR data analysis must consider the time relationship between medical records. Phuoc Nguyen et al. proposed the Deep model [7] which used Convolutional Neural Networks (CNN) to extract features of long sequences in EHR, but CNNs can only capture local feature information. Yu Cheng et. al. represent the long-term EHR data of every patient as a temporal matrix with time on one dimension and event on the other dimension, and then build a CNN structure for extracting phenotypes and perform risk prediction for Congestive Heart Failure (CHF) and Chronic Obstructive Pulmonary Disease (COPD) respectively [25]. Edward Choi et al. proposed Doctor AI [26], which was applied to the task of disease diagnosis and prescription recommendation. Doctor AI was a RNN based temporal model for longitudinal EHRs which maps diagnosis codes, medication codes or procedure codes together into a low dimensional space through multi-hot encoding.

Similarly, for the multi-label classification task of desease diagnoses, Lipton et al. [27] applied RNNs with LSTM hidden units to model varying-length sequences and capture long range dependencies in multivariate time series of clinical measurements in EHR. Further, Choi et al. [28] proposed Med2Vec for the representation learning of medical codes and visits from EHRs, simply using the structure of Multi-Layer Perceptron (MLP). The multiple medical concepts in EHRs such as diagnosis, medication, procedure codes and visits were effectively learned through Med2Vec.

For multi-modal EHR data, Double Core Memory Networks (DCMN) was proposed to integrate information from different modalities of the longitudinal patient data and learn a joint patient representation [29]. In DCMN, two external memory cores compress and store two modalities of sequential data, which interact with each other. In addition to supervised learning, eNRBM (electronic medical records-driven nonnegative restricted Boltzmann machines) [30], Deep Patient [8], Grouped Correlational GAN [31] and other unsupervised representation learning methods also achieved good performances in reconstructing EHRs.

#### Attention in EHR data analysis

Attention mechanism has attracted extensive attentions in deep learning [32]. When human beings observe the environment, it is difficult for them to achieve all aspects, but only pay attention to a few parts of interest to obtain relevant information to construct their own cognition of the environment. The classical attention structure is applied to machine translation tasks, which shows the alignment effect between source language and target language. In 2017, Google proposed Transformer, a translator involving self-attention and multi-head attention, which makes long-distance semantic dependencies and expressions more accurate [33]. A variety of extensions of the attention mechanism have been designed.

In medical research, attention mechanism can simulate the doctor’s inquiry on the patient’s past medical history, by paying more attention to the more closely-related records and attributing higher weights to them. For example, Choi et al. proposed the RETAIN model [34], which designed a Two-level Attention mechanism to learn the weight vectors of the development of the disease and the interaction between diseases respectively. Finally, the patient health status representation is obtained by dot product of these two vectors. In RETAIN, the term ’RET’ refers to REverse Time training, which means that the more recent hospitalization records should get higher attention. Li et al. [20] proposed a Gated Recurrent Unit Networks framework integrating attention mechanism for extracting biomedical events between biotope and bacteria from biomedical literature. Jose et al. [35] conducted heart disease prediction tasks on the CPRD (Clinical Practice Research Datalink) dataset, which once again verified the excellent performance of RETAIN compared with eNRBM, Deep Patient and Deepr [7]. Attention mechanisms are usually closely associated with RNN and its variants, including GRU, LSTM, Bi-LSTM, etc. [36, 37]. HealthATM extracts multifaceted patient information with attentive and time-aware modulars based on a hybrid network composed of both RNN and CNN [38]. The learned representations are then fed into a prediction layer for the risk prediction task.

#### Clinical risk prediction with EHR data

For clinical risk prediction, deep learning approaches also showed competitive performance compared to traditional approaches, e.g. HealthATM was applied in the task of risk prediction of CHF [38]. Zeng et al. [39] designed a concept-based filter and a prediction model to detect breast cancer local recurrence using EHRs. Huang et al. proposed a regularized Stacked Denoising Autoencoder (SDAE) model to stratify clinical risks of Acute coronary syndrome (ACS) patients from a real clinical EHR dataset of 3464 patient samples, and obtained robust and accurate performance [40]. In a recent study, Stephanie L. Hyland et al. used LSTM to develop an early-warning system that provided early identification of patients at risk for circulatory failure by integrating measurements from multiple organ systems [41]. Wanyan et al. introduced the contrastive learning framework with two novel positive sampling strategies (feature-based and attribute-based) and proposed a novel contrastive regularized clinical classification model to predict the mortality risk in real-world COVID-19 EHR data [42].

However, most of previous approaches were proposed for the risk prediction of a single disease, e.g., CHF, COPD, and ACS. For a more general risk prediction task, i.e., hospital mortality of ICU patients, Yu et al. [4] proposed a Multi-Task Recurrent Neural Network based on attention mechanism, which achieved much better recall rate (0.503 vs 0.365) compared with SAPS-II. Nevertheless, the model used only time series measurements of monitoring data based on 24-h observation period, like heart rate, systolic blood pressure and body temperature, while other rich information available in EHR, i.e., the disease and treatment, are still ignored. In this paper, our study shows that AUC (Area Under ROC Curve) and recall rate of mortality risk prediction can be improved by using disease and treatment information in EHR.

In this paper we develop an accurate and clinically interpretable model that predicts hospital mortality for ICU patients using disease and treatment information available in longitudinal EHR. Generally speaking, patient information extracted from EHRs often presents a multi-nested structure, i.e., a patient has multiple longitudinal medical records while each record is composed of multiple diagnoses and prescriptions, as shown in Fig. 1. To fully mine the deep information in EHRs, the following challenges need be addressed:

- The heterogeneity in EHRs hinders from effective extraction of information from EHRs. Heterogeneous data such as diagnoses, prescriptions and other treatments contained in patient records are usually regarded as discrete variables and often have different scales. They should be processed reasonably first, which is the premise of exploring the relationship between them.
- Time dependence always exists in multiple longitudinal medical records of a patient. For example, diabetes and prediabetes are risk factors for cardiovascular disease. Studies have shown that, in the case of the same age, the elderly patients with new onset diabetes have fewer microvascular complications than those with long-term diabetes, that is, the impact of hyperglycemia on human body is time-dependent. Therefore, the model should take into account how to establish time dependence between the longitudinal records of a patient.
- Complex interaction exists not only between diseases, but also between diseases, interventions and treatments. For example, long term chronic hyperglycemia increases microvascular complications such as retinopathy and kidney disease, and lesions in these organs increase the risk of death. Besides, different medical intervention will affect the evolution of the disease, and there are also synergistic or antagonistic effects between drugs.

Aiming at the above challenges, we propose an end-to-end deep learning based mortality risk prediction model for ICU patients, namely DeepMPM, which can automatically extract high-quality representations from heterogeneous, multi-nested and longitudinal EHRs. We introduce a Two-level Attention Long-Short Term Memory Neural Network (LSTM) simulating doctor’s inquiry behavior to obtain information that assist in evaluating the current status of patients from their longitudinal medical records. The LSTM module generates two weight vectors, respectively focusing on the interactions between disease development and treatment. Finally, one full connected layer with Softmax classifier is trained to output the mortality risk probability of the patient. The contributions of this study are as follows:

- Rather than the monitoring data or risk factors, DeepMPM leverages the discrete ICD-9 code (International Statistical Classification of Diseases and Related Health Problems 9th Revision) [43] and DRGs code (Diagnosis Related Groups) [44] in EHR which contain more rich information. Experiment results on MIMIC III database [45] demonstrates that with the disease and treatment information deep learning approaches can achieve significantly better accuracy than conventional approaches of mortality risk prediction.
- In contrast to mortality risk prediction methods for a single disease, we show the benefits of using EHR from patients with multiple diseases and different conditions to predict the mortality risk. A comparison experiment indicates that DeepMPM can successfully model the complex correlation between diseases to achieve better representation learning of disease and treatment, so as to improve the accuracy of mortality prediction.
- A case study shows that the framework of DeepMPM offers the potential to provide users with insights into EHR data and model behavior in mortality prediction task, respectively. First, the encoder can provide a global view of the feature correlation in EHR. Second, for each mortality risk prediction of one patient, the two-level attention LSTM module generates the corresponding weight vectors which reflect the visit-level importance of the longitudinal records and variable-level importance of features respectively.

The rest of the paper is organized as follow. In “Methods” section we describe the proposed framework. In “Results” section we present the experiments under different settings, and demonstrate the merits of the new framework. In “Discussion” section we give a case study to discuss the model interpretability. “Conclusions” section summarizes the work.

## Methods

### Overview of the framework

**Fig. 2:**
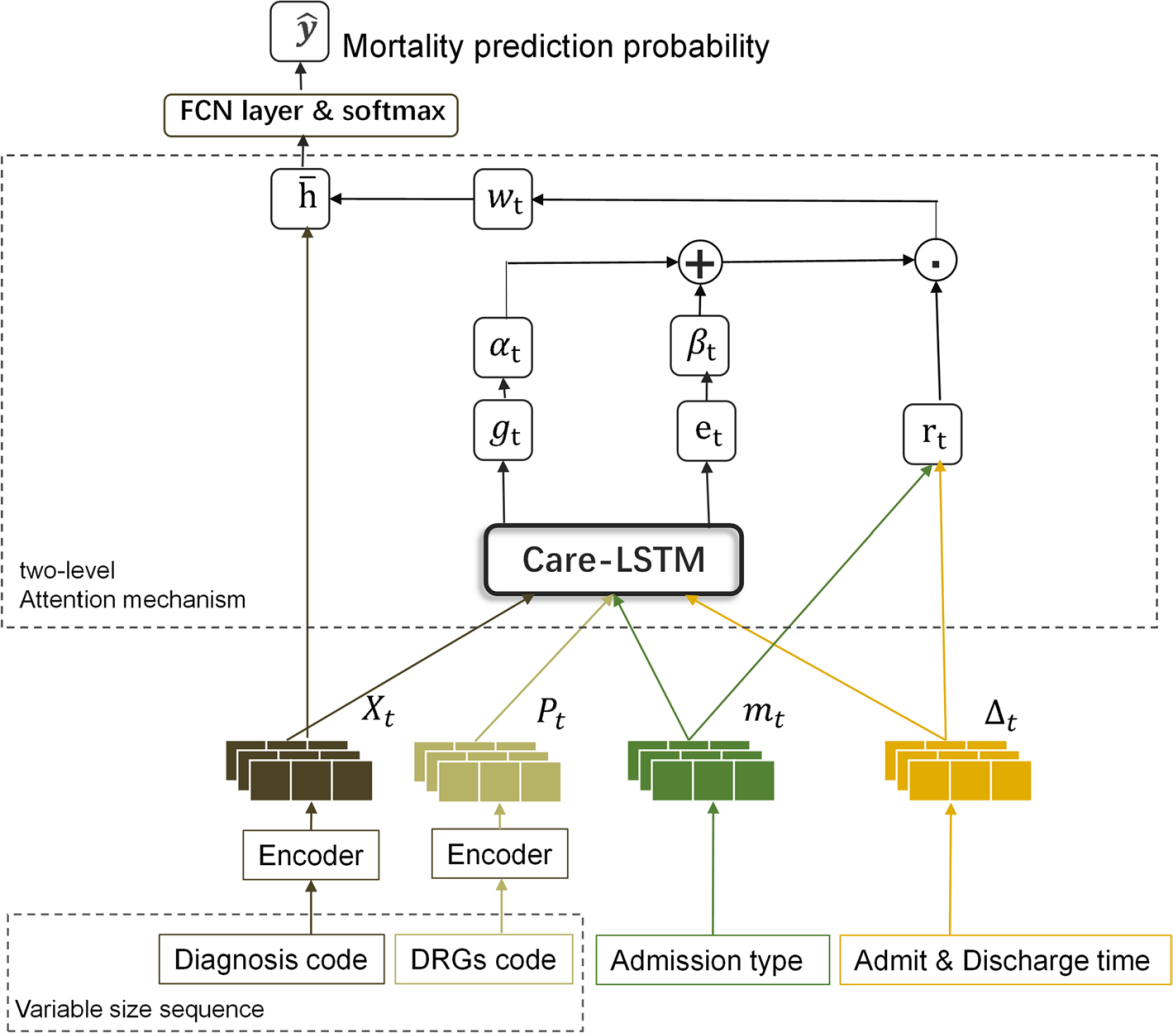
The framework of DeepMPM: a mortality risk prediction model using two-level attention mechanism and integrating multiple data types

DeepMPM is an end-to-end deep model for predicting the mortality risk of patients using longitudinal EHRs. Figure 2 depicts the overall framework of DeepMPM. The EHRs used in the model can include diagnoses codes, treatment and intervention codes, hospitalization type and admission/discharge time, etc. In this paper, we use the MIMIC III database [45], and use DRGs (Diagnosis Related Groups) codes to represent the medical treatment and intervention, considering that the DRGs codes also contain rich information of diseases and are convenient to use. The detailed information of the database is introduced in “Data description” section. The notations used throughout this paper are summarized in Table 1. DeepMPM mainly consists of three steps:

**Table 1:** Table of notations

Notation	Meaning
*D*	Diagnoses codes set, $D=\{d_{1}, d_{2}, \ldots , d_{k}\}$
*L*	DRGs codes set, $L=\{l_{1}, l_{2}, \ldots , l_{s}\}$
$X_t$	Representation vector of diagnosis
$P_t$	Representation vector of treatment
$x_t$	Diagnoses codes of a record, $x_t \in \{ 0,1\}^{|D|}$
$p_t$	DRGs codes of a record, $p_t \in \{ 0,1\}^{|L|}$
$W_{xemb}$	Weight of embedding layer for diagnoses codes
$W_{pemb}$	Weight of embedding layer for DRGs codes
$f_t$	Forget gate of LSTM at time step *t*
$W_f$	Weight of the forget gate of LSTM
$i_t$	Input gate of LSTM at time step *t*
$W_i$	Weight of the input gate of LSTM
$\tilde{C_t}$	Candidate cell state of LSTM at time step *t*
$C_t$	Cell state of LSTM at time step *t*
$o_t$	Output gate of LSTM at time step *t*
$W_o$	Weight of the output gate of LSTM
$h_t$	Hidden state of LSTM at time step *t*
$m_t$	Type of hospitalization
$q_t$	Hospital stay vector
$U_i$	Weight of $h_{t-1}$ in the input gate of Care-LSTM
$U_f$	Weight of $h_{t-1}$ in the forget gate of Care-LSTM
$P_f$	Weight of $P_{t-1}$
$Q_f$	Weight of $q_{\Delta _{t-1:t}}$
$q_{\Delta _{t-1:t}}$	Hospital stay during $\Delta _{t-1:t}$
$\Delta _{t-1:t}$	Adjacent hospital stay intervals
$U_o$	Weight of $h_{t-1}$ in the output gate of Care-LSTM
$P_o$	Weight of $P_t$
$g_t$	Output of the hidden layer of Care-LSTM at time step *t*
$\alpha _t$	Variable-level weight vector, $\alpha _t \in [0,1]$
$W_\alpha ^T$	Weight matrix in attention module
$e_t$	Output of the hidden layer of Care-LSTM at time step *t*
$\beta _t$	Visit-level weight vector, $\beta _t\in [-1,1]$
$W_\beta$	Weight matrix in attention module
$r_t$	Harmonic weight coefficient
$w_t$	Final weight vector of the two-level attention module
${\bar{h}}$	Patient health status vector

**Fig. 3:**
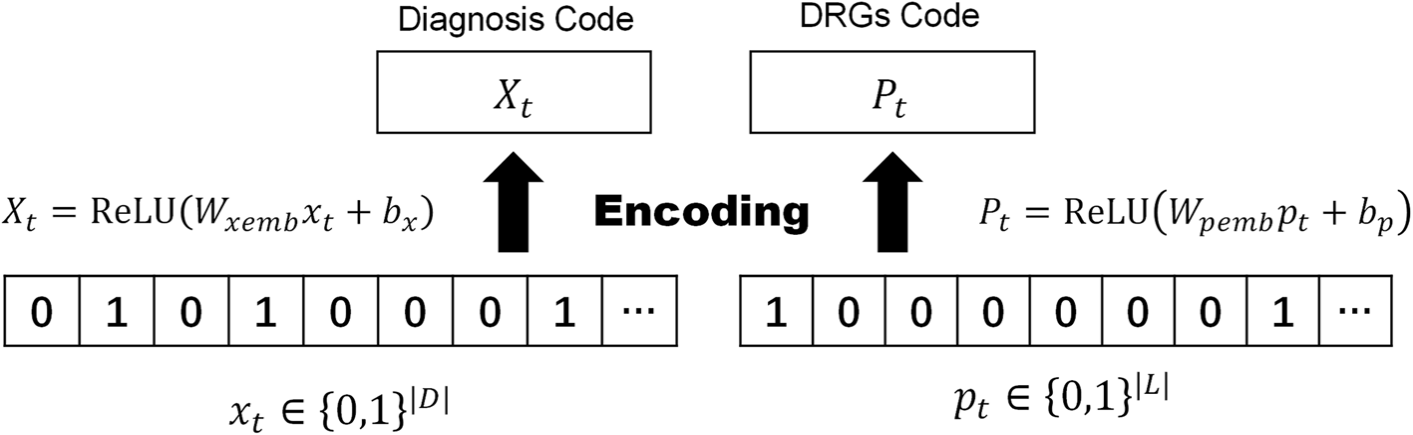
The encoder in DeepMPM: the varying-length sequence of diagnoses and DRGs codes are represented as equal-length vectors in a specific vector space

**Fig. 4:**
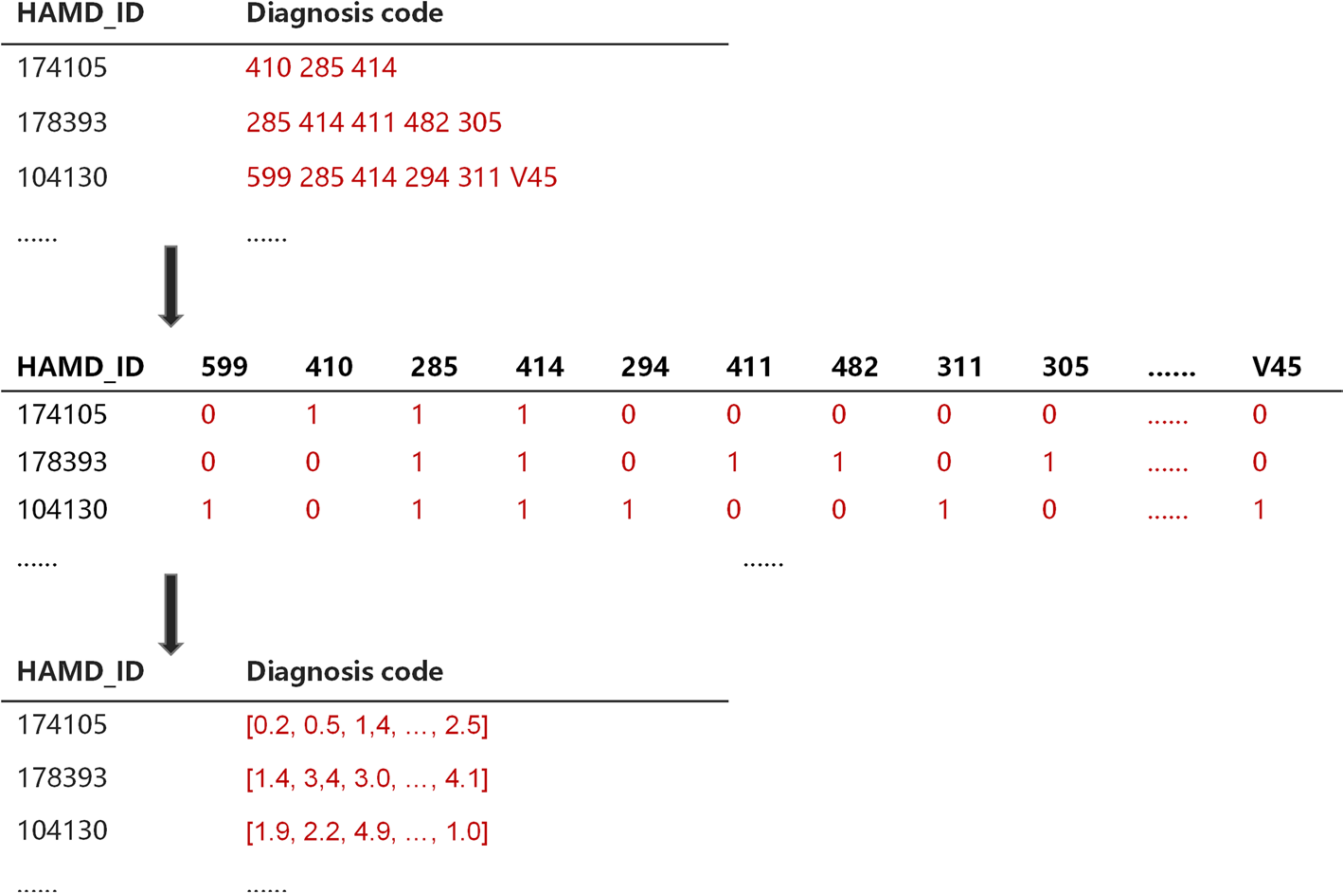
DeepMPM’s representation learning on disease codes: HAMD_ID represents the ID of diagnosis record, and each record has corresponding varying-length coding sequence. Firstly, all sequences are represented as a binary matrix, and then mapped to a specific vector space, and the varying-length sequence is transformed into multi-dimensional equal-length non-negative vector

- Step 1: An encoder model is used to map the discrete diagnoses codes and DRGs codes to a continuous vector space, and the corresponding representation vectors $X_t$ and $P_t$ are obtained respectively. As shown in Fig. 3, the encoder contains a single layer structure: let $D=\{d_{1}, d_{2}, \ldots , d_{k}\}$ denotes the diagnoses codes set, and $L=\{l_{1}, l_{2}, \ldots , l_{s}\}$ denotes the DRGs codes set. The sequence of diagnoses codes or DRGs codes of each record can be represented by a binary vector. Then we can use $x_t \in \{ 0,1\}^{|D|},p_t \in \{ 0,1\}^{|L|},t = 1,2,\ldots ,T$ to represent *T* hospitalization records (including ICU and non-ICU hospitalization records). Here we use a simplified version of Med2vec [28] based on Skip-gram model. Intuitively, the skip-gram model predicts other codes that appear in the context by giving the input code, and can learn the co-occurrence between different codes, so that the representation vectors of related codes are similar, and the representation vectors of unrelated codes are different. Finally, the two binary vectors are respectively embedded into high dimensional space by the encoder as follows,
  1 $$\begin{aligned} X_{t}= & {} {\text {ReLU}}\left( W_{xemb} x_{t}+b_{x}\right) \end{aligned}$$
  2 $$\begin{aligned} P_{t}= & {} {\text {ReLU}}\left( W_{pemb} p_{t}+b_{p}\right) \end{aligned}$$
  where $W_{xemb}\in R^{d\times |D|},W_{pemb} \in R^{d\times |L|},b_x \in R^d,b_p \in R^d$. Here the adoption of multi-hot encoding avoids the sparsity of one-hot encoding, and improves the computational efficiency. An illustration example of the process is shown in Fig. 4.

- Step 2: A two-level attention mechanism combined with LSTM is designed to realize the representation learning of patient health status. The visit-level attention mechanism focuses on the development and evolution of the disease, and explores the relationship between diagnosis and treatment at multiple time points, because the current health status of patients is closely related to the past medical history. The variable-level attention mechanism focuses on the interaction of multiple diseases or treatment within the same record, because there are often concurrent relations between multiple diseases and synergistic effects of drugs or therapeutic interventions. The two weight vectors of two-level attention module, namely $\alpha _t$ and $\beta _t$, are respectively obtained using modified Care-LSTM [46] which combined diagnoses, medications, hospitalization type, time interval and other variables. Finally, the total weight coefficient $w_t$ is obtained by adjusting a harmonic weight coefficient $r_t$, and the final state vector ${\bar{h}}$ is obtained after weighted averaging with the diagnosis representation vector of each record.
- Step 3: Predict the risk probability by using the full connected layer and softmax function. Here we use cross entropy to calculate the classification loss as follows:
  3 $$\begin{aligned} \begin{aligned} \text{ Loss }=-\frac{1}{N} \sum _{n=1}^{N} \frac{1}{T^{(n)}} \sum _{i=1}^{T^{(n)}}(y_{i}^{T} \log ({\hat{y}}_{i})+(1-y_{i})^{T} \log (1-{\hat{y}}_{i})) \end{aligned} \end{aligned}$$
  where *N* is the total number of samples, *n* refers to the *n*th patient sample, $n = 1,2,\ldots ,N$. $T^{(n)}$ refers to the total number of hospital records for the *n*-th patient sample. $y_i \in \{0, 1\}$ is the value of the death variable in the *i*th hospitalization record of the *n*th sample, where 0 means discharged and 1 means death.

### Modified care-LSTM

**Fig. 5:**
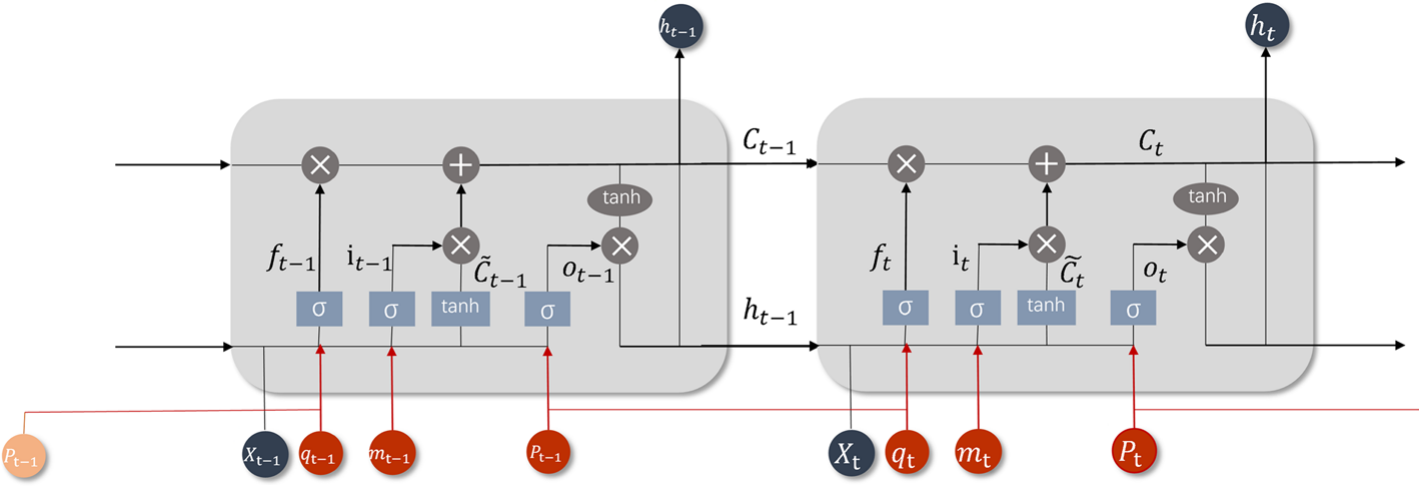
Modified Care-LSTM: the input units marked in red are the parts different from the standard LSTM

RNN allows the internal circulation of information, which can ensure that the previous information is used in each step of calculation, so as to connect the previous information with the current task, which is suitable for sequence data. However, RNN has difficulties in learning long-term dependencies from data, while LSTM, a special kind of RNN, capable of learning long-term dependencies. Instead of having a single neural network layer, each neuron in LSTM has four interacting layers, i.e., a memory cell and three gates. Equation 4 - Eq. 9 describe the operation principle of LSTM. The core is the cell state $C_t$, which runs straight down the entire chain as shown in Fig. 5. Attributed to cell state, even information from a long time ago can flow through the whole network. The cell state is controlled by three gates: forget gate $f_t$, input gate $i_t$ and output gate $o_t$. The forget gate is a sigmoid layer which specifies how much information in $C_{t-1}$ to preserve by looking at a vector determined by $x_t$ and $h_{t-1}$. Another sigmoid layer called the input gate determines the information to be updated by combining the candidate cell states $\tilde{C_t}$. Then, the forget gate and input gate are used together to update the cell state of the current time step. Finally, a vector ranging in $[-1,1]$ is obtained by passing the cell state $C_t$ through a tanh layer, which is then multiplied by the output gate to determine the final output of the neuron.

4 $$\begin{aligned} f_t & {} = {\text {sigmoid}}\left( W_f\cdot \left[ x_t,h_{t-1}\right] +b_f\right) \end{aligned}$$

5 $$\begin{aligned} i_t & {} = {\text {sigmoid}}\left( W_i\cdot \left[ x_t,h_{t-1}\right] +b_i\right) \end{aligned}$$

6 $$\begin{aligned} \tilde{C_t} & {} = {\text {tanh}}\left( W_c\cdot \left[ x_t,h_{t-1}\right] +b_c\right) \end{aligned}$$

7 $$\begin{aligned} C_t & {} = f_t*C_{t-1}+i_t*{\tilde{C_t}} \end{aligned}$$

8 $$\begin{aligned} o_t & {} = {\text {sigmoid}}\left( W_o\cdot \left[ x_t,h_{t-1}\right] +b_o\right) \end{aligned}$$

9 $$\begin{aligned} h_t & {} = o_t*{\text {tanh}}(C_t) \end{aligned}$$

In DeepMPM, we design a modified version of Care-LSTM which was first proposed in the DeepCare model [46]. Compared with the standard LSTM, in addition to $X_t$, we also include the treatment vector $P_t$, the type of hospitalization $m_t$, and the hospital stay vector $q_t$ composed of adjacent hospital stay intervals $\Delta _{t-1:t}$. Figure 5 shows the structure of modified Care-LSTM obtained by adding new variables (shown in red) on the basis of the standard LSTM, which enables the attention mechanism to measure more information when assigning weights. Specifically, Eqs. 10–13 describe the operation principle of modified Care-LSTM. Each time step refers to a record of the patient. The output gate is intervened by $P_t$ at the current time, while the forget gate is intervened by $P_{t-1}$ of the previous time step. The hospital stay vector $q_t$ is also added into the forget gate, where the adjacent time interval $\Delta _{t-1:t}$ = current admission time – last hospitalization discharge time (days). The adjacent time intervals of patients’ visits have a large span. Inspired by parameter settings of the original Care-LSTM, we adopted three different time scales (60, 180, and 360 days) to obtain three levels of time interval representations, which tries to represent richer semantic information of time interval from different scales. The weight coefficient of hospitalization type $1/m_t$ is included in the input gate. If it is emergency hospitalization, the weight is heavy, otherwise the weight is small.

10 $$\begin{aligned} i_t & {} = \frac{1}{m_t}\cdot {\text {sigmoid}}\left( W_i\cdot X_t+U_i\cdot h_{t-1}+b_i\right) \end{aligned}$$

11 $$\begin{aligned} f_t & {} = {\text {sigmoid}}(W_f\cdot X_t+U_f\cdot h_{t-1}+P_f\cdot P_{t-1}+Q_f\cdot q_{\Delta _{t-1:t}}+b_f) \end{aligned}$$

12 $$\begin{aligned} q_t & {} = \left[ \frac{\Delta _{t-1:t}}{60},\frac{\Delta _{t-1:t}}{180},\frac{\Delta _{t-1:t}}{365}\right] \end{aligned}$$

13 $$\begin{aligned} o_t & {} = {\text {sigmoid}}\left( W_o\cdot X_t +U_o\cdot h_{t-1}+P_o\cdot P_t+b_o\right). \end{aligned}$$

### The two-level attention mechanism

When applied to the field of medical data research, the attention mechanism can simulate doctors’ comprehensive analysis of longitudinal EHR of the patient. In DeepMPM, we design a two-level attention mechanism that are different from RETAIN [34]. In RETAIN, a standard GRU network was used and the model only considered the diagnosis and the length of hospital stay. Moreover, the weight vector is obtained by reverse time training, which assumes that more attention should be paid to recent records.

Unlike RETAIN, DeepMPM takes into account the influence of time interval between visits, which makes the time interval parameterized in the expression of forget gate, and participates in the weighting of the longitudinal records. Further, DeepMPM does not adopt the reverse time training strategy, since we find that the it reduced the accuracy in preliminary experiment, and the assumption behind RETAIN does not always hold. The reason may lies in the fact that for patients with chronic diseases, a certain disease may follow the patient for many years, which has potential threat to the patient’s health, and becomes an important factor causing the deterioration of the health status. Instead, we reduce the impact of non-emergency and long-term records with a harmonic weight coefficient, as a supplement to the weight vectors learned from the two-level attention mechanism which tries to discover the complex relationships. .

In DeepMPM, the visit-level weight vector $\alpha _t$ measures the relevance of longitudinal records at different time points, which actually reflects the development and evolution of the diseases. The scalars $\alpha _1,\ldots , \alpha _t$ are the visit-level attention weights that measure the importance of each visit embedding $v_1,\ldots , v_t$. Specifically, Eqs. 14–15 show the calculation of $\alpha _t$ using modified Care-LSTM and softmax function.

14 $$\begin{aligned} g_t = & {} {\text {Care-LSTM}}\left( \left[ X_t;P_t;m_t;\Delta _{t-1:t}\right] \right) \end{aligned}$$

15 $$\begin{aligned} \alpha _t = & {} {\text {softmax}}\left( W_\alpha ^Tg_t+b_\alpha \right) \end{aligned}$$

where $g_t \in R^p$ denotes the output of the hidden layer of Care-LSTM at time step *t*, the parameters $W_\alpha \in R^p, b_\alpha \in R,\alpha _t \in [0,1]$.

The variable-level weight vector $\beta _t\in R^d$ measures the internal relationship within the same record, which actually reflects the interaction of different diseases and different treatments. The vectors $\beta _1,\ldots , \beta _t$ are the variable-level attention weights that measure each variable’s importance of the visit embedding $v_{1,1}, v_{1,2},\ldots , v_{1,d},\ldots , v_{t,1}, v_{t,2},\ldots , v_{t,d}$. Specifically, Eqs. 16 and 17 show the calculation of $\beta _t$ using Care-LSTM and the tanh function. Note that $\beta _{t,d}\in [-1,1]$, and a negative value indicates a suppression relation between diseases and treatments while a positive value indicates the synergistic effect between them.

16 $$\begin{aligned} e_t & {} = {\text {Care-LSTM}}\left( \left[ X_t;P_t;m_t;\Delta _{t-1:t}\right] \right) \end{aligned}$$

17 $$\begin{aligned} \beta _t & {} = {\text {tanh}}(W_\beta e_t+b_\beta ) \end{aligned}$$

where $e_t\in R^q$ denotes the output of the hidden layer of Care-LSTM at time step *t*, and $W_\beta \in R^{d\times q},b_\beta \in R^d$. Taking into account the time dependence, Eq. 18 defines a harmonic weight coefficient $r_t \in {R^d}$ as a supplement to the weight vectors $\alpha _t$ and $\beta _t$ as follows,

18 $$\begin{aligned} r_t = \left[ m_t+\log (1+\Delta _{t:T})\right] ^{-1} \end{aligned}$$

where $\Delta _{t:T}$ is the length of days between the *t-th* admission and the last discharge. We use this weight to reduce the impact of long-term and non-emergency hospitalization records, hence we get the final weight vector $w_t\in R^d$ as follows,

19 $$\begin{aligned} w_t=r_t\odot \frac{(\alpha _t+\beta _t)}{2} \end{aligned}$$

where $\odot$ represents the Hadamard product or element-wise product. Extend the scalar $\alpha _{t}$ to a vector of length *d*. Then the patient health status representation vector ${\bar{h}}$ is obtained by weighted averaging of $X_t$ as follows,

20 $$\begin{aligned} {\bar{h}}= & {} \left( \sum \limits _{t=t1}^T{w_t}\cdot X_t\right) / \sum \limits _{t=t1}^T{w_t} \end{aligned}$$

21 $$\begin{aligned} P({\hat{y}}|[X_t;P_t;m_t;\Delta t])= & {} {\text {softmax}}(FCN({\bar{h}})) \end{aligned}$$

Finally, the mortality risk of patient is derived using Eq. 21, with *FCN* representing the full connected layer.

### Dataset

**Fig. 6:**
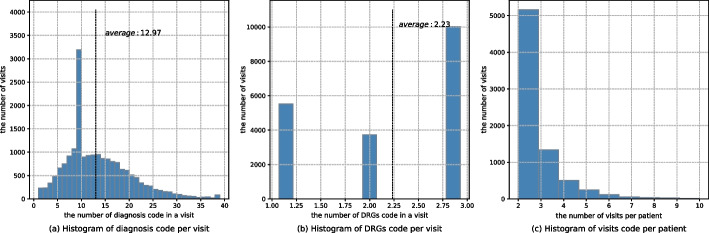
**a** The distribution of ICD-9 codes in a single record. The average value is 12.97. **b** The distribution of DRGs codes in a single record. The average value is 23. **c** The distribution of visit numbers of each patient. The average value is 2.57

The experimental data used in this paper are from the MIMIC III (Medical Information Mart for Intensive Care) database, which is a large and freely accessible database comprising medical records relating to patients admitted to critical care units at a tertiary care hospital [45]. The records includes vital signs, medications, laboratory measurements, observations and notes, procedure codes, diagnostic codes, hospital length of stay, survival data, etc.

In data cleaning, patients with a single record or more than 10 medical records were excluded. DeepMPM takes time into account as a training factor, so training data with only one hospitalization record cannot provide effective information on time to disease treatment. On the other hand, the records of patients with more than 10 hospitalizations often span decades, and disease evolution over decades is complex and may not be correlated, and it is likely to cause matrix sparseness, affecting model training results. After statistical analysis of the original patient’s medical records, we observed that 10 is an appropriate threshold. Moreover, excluding the patient samples with more than 10 hospitalization records will not affect the proportion of positive and negative samples. After data cleaning, the positive-to-negative ratio of samples is 1.074:1.

After data cleaning, a total of 7491 patients were selected from MIMIC III, with a total of 19,265 valid records. In our study, multiple records of each patient include diagnostic code, DRGs (Diagnosis Related Groups) code, hospitalization type, admission/discharge time, DOD (date of death. If the patient is alive, it is indicated by null value).

**Table 2:** EHR data description

	Item	Value
The number of patients	–	7491
The number of visits	–	19,265
positive samples/negative samples ratio	–	1.074:1
Avg. number of visits per patient	–	2.57
Diagnoses code	The number of code groups	931
	Avg. number of codes per visit	12.97
	Max. number of codes per visit	39
DRGs code	The number of code groups	1406
	Avg. number of codes per visit	2.23
	Max. number of codes per visit	3

In MIMIC III, the diagnostic code is represented with ICD-9 code (International Statistical Classification of Diseases and Related Health Problems 9th Revision). In order to avoid overfitting, the first three bits of all ICD-9 codes are intercepted to represent the disease. The DRGs is a kind of patient classification scheme. It comprehensively considers the main diagnosis and treatment methods of patients according to the discharged medical records, and divides the medical records with similar disease complexity and cost into the same group according to the individual signs, age, complications and accompanying diseases, and distinguishes them by different digital codes. Considering that DRGs is a comprehensive summary of the patient’s symptoms, conditions and treatment, it is used to represent the medical treatments and intervention. Our preliminary experiments also showed that using DRGs codes could improve the performance.

There are four admission types in MIMIC III data, i.e., elective, urgent, newborn and emergency. We regrouped them into “emergency (including emergency and urgent)” and “non-emergency (including elective and newborn)”. Since the time of admission/discharge has been desensitized in MIMIC III data, we obtain the length of hospital stay from these two items. DOD represents the time of hospital death and is transformed to the binary classification label of mortality risk. The hospital death is marked as positive (“1”) while the survivors is marked as negative (“0”).

Table 2 displays the data statistics. A total of 931 ICD-9 codes and 1406 DRGs codes are included in the data. The ratio of positive to negative samples (P/N ratio) is 1.074:1, indicating that the experimental data is balanced. The average number of records per patient is 2.57. There are 12.97 diagnosis codes and 2.23 DRGs codes per record. The data distribution is shown in Fig. 6.

### Baseline methods

In order to test the effectiveness of DeepMPM, we use the following five deep learning models for comparison:

- RNN: a standard GRU without attention mechanism. The learning rate is 0.05, the size of the disease representation vector is 32, and the hidden layer size is 64.
- Multi-task learning [4]: a multi-task RNN prediction model with attention mechanism. Since the data used in this paper is different from the ICU physiological monitoring data in [4], here we use the same encoder with DeepMPM which contains 32 hidden units, and use a GRU which contains 32 hidden units as the decoder. The learning rate is set to 0.1.
- LSTM-NN: the classification module in [4], which uses LSTM for feature learning and outputs the prediction of inpatient mortality through two full connected layers. The encoder and decoder are the same as the multi-task learning module, and the learning rate is set to 0.05.
- RETAIN [34]: using two-level attention mechanism and reverse time training strategy. The learning rate is set to 0.1. The size of the hidden model was 32, the size of the representation vector is 32, and the hidden layer size is 64.
- DeepCare [46]: the authors proposed three methods for characterizing varying-length records: taking the maximum, taking the average and summation. In the experiment, we find the best pooling method is to take the average value to form the representation of equal length records. The size of representation vectors are 32, the size of hidden layer is 64, and the learning rate is 0.01.
- DeepMPM-w/o-$\beta$: a variant of DeepMPM that removes the variable-level attention mechanism, that is, only retains the visit-level attention mechanism. The final weight $w_{t}$ is obtained by $w_t=r_t\odot \alpha _{t}$. The size of the representation vector is 32, and the hidden layer size is 64.

We implement DeepMPM in Theano 1.0.5. All models are running on Python 3.7.10, with GTX 1080 GPU, 96GB RAM, and 3.50GHz i7-7800X CPU.

In the Table 3, we compare the differences between the baseline models and the proposed method. All the baseline methods and the proposed methods are based on the RNN architecture. Among them, RETAIN believes that the recent hospital admission records are more helpful for diagnosis, so the disease history records are entered in reverse time order. Muti-task Learning, LSTM-NN, RETAIN, DeepMPM-w/o-$\beta$ and the proposed DeepMPM introduce the attention mechanism. Among them, RETAIN and the proposed DeepMPM adopt the two-level attention mechanism, including visit-level and variable-level attention mechanisms. DeepMPM-w/o-$\beta$ only retains the visit-level attention mechanism for comparison with DeepMPM.

**Table 3:** Comparison of the characteristics of the baseline methods with DeepMPM

Model	RNN architecture	Reverse time training	Attention mechanism	Visit-level attention	Variable-level attention
RNN	$\surd$	$\times$	$\times$	$\times$	$\times$
Multi-task Learning	$\surd$	$\times$	$\surd$	$\times$	$\times$
LSTM-NN	$\surd$	$\times$	$\surd$	$\times$	$\times$
RETAIN	$\surd$	$\surd$	$\surd$	$\surd$	$\surd$
Deepcare	$\surd$	$\times$	$\times$	$\times$	$\times$
DeepMPM-w/o-$\beta$	$\surd$	$\times$	$\surd$	$\surd$	$\times$
DeepMPM	$\surd$	$\times$	$\surd$	$\surd$	$\surd$

**Fig. 7:**
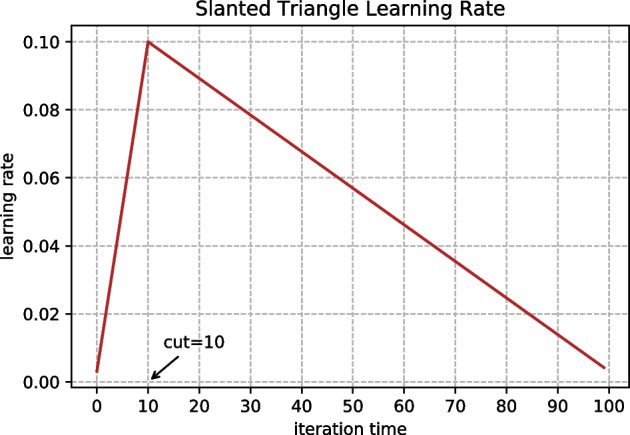
Slanted triangle learning rate. The curve of learning rate is similar to a triangle, and its expression is shown in Eqs. 22–24, where *T* is the total number of training iterations, $cut_ Frac$ is the percentage of rising segment to the total number of iterations, and ratio determines the lowest value of learning rate. In the experiment, we set $cut_Frac=0.1$, $ratio=32$, $\eta _{max}=0.1$

For fair comparison, the same encoder is used to obtain the representation vector of disease and DRGs for all the models. In model training, the small batch training method is used, the batch size is 80 and the iteration times is 100. For parameter tuning, five-fold cross-validation grid search is applied for all the models. Adadelta algorithm [47] is used for model training, and the attenuation coefficient $\rho =0.1$. Besides, STLR (Sloped Triangular Learning Rates) [48] is adopted, as shown in Fig. 7. In order to avoid overfitting, L1 and L2 regularization are added to the loss functions, and the regularization coefficient is 0.0001. The Dropout technique is adopted to train the neural networks, and the activation value $p=0.8$.

22 $$\begin{aligned} cut= & {} T\cdot cut\_frac \end{aligned}$$

23 $$p = {\text{ }}\left\{ {\begin{array}{*{20}l} {t/cut,\quad t < cut} \hfill \\ {1 - \frac{{t - cut}}{{cut \cdot \left( {\frac{1}{{cut\_frac}} - 1} \right)}},\quad {\text{otherwise}}} \hfill \\ \end{array} } \right.$$

24 $$\begin{aligned} \eta _t= & {} \eta _{max}\cdot \frac{1+p\cdot (ratio-1)}{ratio}. \end{aligned}$$

### Evaluation metrics

In order to comprehensively evaluate the performance of each model, we use AUC (Area Under ROC Curve), precision rate, recall rate and F1-score as the evaluation metrics [49].

25 $$\begin{aligned}&Precision=\frac{TP}{TP+FP} \end{aligned}$$

26 $$\begin{aligned}&Recall=\frac{TP}{TP+FN} \end{aligned}$$

27 $$\begin{aligned}&F_1=\frac{2\times Precision\times Recall}{Precision+Recall} \end{aligned}$$

where *TP*, *FP* and *FN* represent the number of true positive, false positive, and false negative, respectively.

## Results

**Table 4:** The results of the performances of different models

Model	AUC	Precision	Recall	F1-score
RNN	0.8318 ± 0.0102	0.7392 ± 0.0340	0.7571 ± 0.0408	0.7505 ± 0.0139
Multi-task learning	0.6330 ± 0.0084	0.6130 ± 0.0245	0.5808 ± 0.1439	0.5868 ± 0.0674
LSTM-NN	0.8326 ± 0.0087	0.7562 ± 0.0289	0.7508 ± 0.0519	0.7621 ± 0.0148
RETAIN	0.8268 ± 0.0081	0.7592 ± 0.0103	0.7788 ± 0.0091	0.7687 ± 0.0089
Deepcare	0.7876 ± 0.0098	**0.7858** ± 0.0264	0.7707 ± 0.0458	0.7782 ± 0.0147
DeepMPM-w/o-$\beta$	0.8435 ± 0.0073	0.7685 ± 0.0210	0.7759 ± 0.0490	0.7710 ± 0.0177
DeepMPM	**0.8501** ± 0.0076	0.7700 ± 0.0306	**0.7987** ± 0.0538	**0.7824** ± 0.0153

The overall best result is given in bold font

### Experiment I: performances comparison of different methods

Table 4 records the four evaluation metrics of each model, which demonstrates that DeepMPM outperforms other methods. It is worth noting that all other methods achieved significantly better recall rate and precision rate than the Multi-task Learning model (0.5808 and 0.6130) just using time series measurements of monitoring data based on 24-h observation period. Although DeepMPM achieves a lower precision (0.7700) than DeepCare (0.7858), it has the best performance in other metrics. The reason is that it performs much better than other methods in recall rate (0.7987). Generally speaking, the metric of recall is more important in the mortality risk prediction, since a larger recall means the model can identify more ICU patients with high mortality risk. DeepCare uses a relatively rough method to allocate the weight of each record according to the type of hospitalization and time interval, which may be too simplistic. Long-term records can also provide important information for judging the patient health status and planning treatment. In addition, even if a patient is admitted to hospital in a non-emergency way, his/her condition may deteriorate during hospitalization, and ignoring this will lead to serious consequences. Compared with DeepCare, RETAIN use a two-level attention mechanism to explore the potential relationship between records, however, it adopts reverse time training strategy and ignores integrating multiple variables in the model. For the multi-task learning method, it is concluded in [4] that it would achieve better prediction effect than single task learning (LSTM-NN) or separate learning. However, whether this conclusion holds or not seems to depend on different data types. On the data used in is paper, it does not hold, either. DeepMPM-w/o-$\beta$ is a version that removes the variable-level attention mechanism. From the experimental results, the lack of variable-level attention mechanism leads to the weakening of model performance.

### Experiment II: why make mortality prediction using the whole EHR dataset

In this section, we show the benefits of using EHR from patients with multiple diseases and different conditions to predict the mortality risk. For a specific disease that can directly cause death, e.g., Congestive Heart Failure (CHF), a natural question is, do we just need to use all the records that contains that disease to predict the mortality risk? In other words, are those EHRs that are not associated with that disease helpful for mortality prediction? In order to investigate the effectiveness of these seemingly “unrelated” EHRs in predicting the mortality risk for a particular disease, we conducted the following comparative experiments: First, we hold out a test set which contains only the EHR records related to a specific disease, e.g., CHF. Then we select two groups of records from the remaining dataset as the training set respectively. Group I only contains the records of patients with that disease, while Group II contains all the remaining records. Two mortality prediction models are then trained using DeepMPM respectively, with the same parameter setting and tuning strategy as Experiment I. Table 5 displays the average results of the four evaluation metrics in five-fold cross validation experiments of two high-risk diseases, i.e., CHF and Diabetes, respectively. The sample size of CHF patients is 3285, while the P/N ratio is 1.808:1. The sample size of Diabetes patients is 2705, while the P/N ratio is 1.3:1.

**Table 5:** The performances on the same test set of DeepMPM trained with different training sets

Training set description	AUC	Precision	Recall	F1-score
Group I: all of patients were diagnosed with CHF	0.7593 ± 0.0473	0.7533 ± 0.0378	0.8419 ± 0.0219	0.7853 ± 0.0145
Group II: Containing patients weren’t diagnosed with CHF	**0.8239** ± 0.0451	**0.7984** ± 0.0098	**0.8428** ± 0.0129	**0.8127** ± 0.0503
Group I: all of patients were diagnosed with diabetes	0.7468 ± 0.0255	0.7244 ± 0.0416	0.7184 ± 0.0214	0.6913 ± 0.0087
Group II: containing patients weren’t diagnosed with diabetes	**0.8014** ± 0.0418	**0.7516** ± 0.0281	**0.7773** ± 0.0573	**0.7562** ± 0.0151

The overall best result is given in bold font

Why can the prediction performance be improved by adding records “unrelated” to the disease? Taking CHF as an example, CHF is a kind of clinical syndrome in which cardiac output cannot meet the needs of metabolism, blood perfusion of tissues and organs is insufficient, and pulmonary or systemic congestion occurs at the same time. It is a clinical syndrome when various heart diseases develop to the serious stage. There are many risk factors for CHF, such as myocardial diastolic dysfunction, mainly referring to diastolic dysfunction, common in hypertension and left ventricular hypertrophy. Other factors include infection, ventricular afterload, arrhythmia and so on. Gottdiener et al. [50] studied and analyzed the cardiovascular data of 5625 elderly people over 65 years old in four regions of the United States. These elderly people all had CHF risk factors. During the average 5.5 years follow-up, 597 people developed CHF. The study found that the high risk factors included hypertension, atherosclerosis, diabetes and other heart diseases, and the incidence rate of elderly men was higher. After statistical analysis of the MIMIC III data used in this study, we find that both CHF patients and Non-CHF patients have a high probability of accompanied by hypertension, heart fibrillation, coronary atherosclerosis, acute renal failure, diabetes and other diseases, so Non-CHF patients may also be the “potential candidates” of CHF. Therefore, the model trained with the data that includes patients who have not been diagnosed with CHF can also learn the characteristics highly related to CHF patients, and may learn extra information missing from CHF records, such as similar medication or treatment methods. The same is true for Diabetes.

Therefore, as long as appropriate methods (such as deep learning) are used, the complex correlation between diseases can be fully utilized to achieve better representational learning of the disease and treatment, so as to improve the performance of mortality prediction. On the other hand, it also indicates that the mortality risk identified by DeepMPM is not aimed at a single disease, but comprehensively reflects the overall health status of ICU patients.

**Fig. 8:**
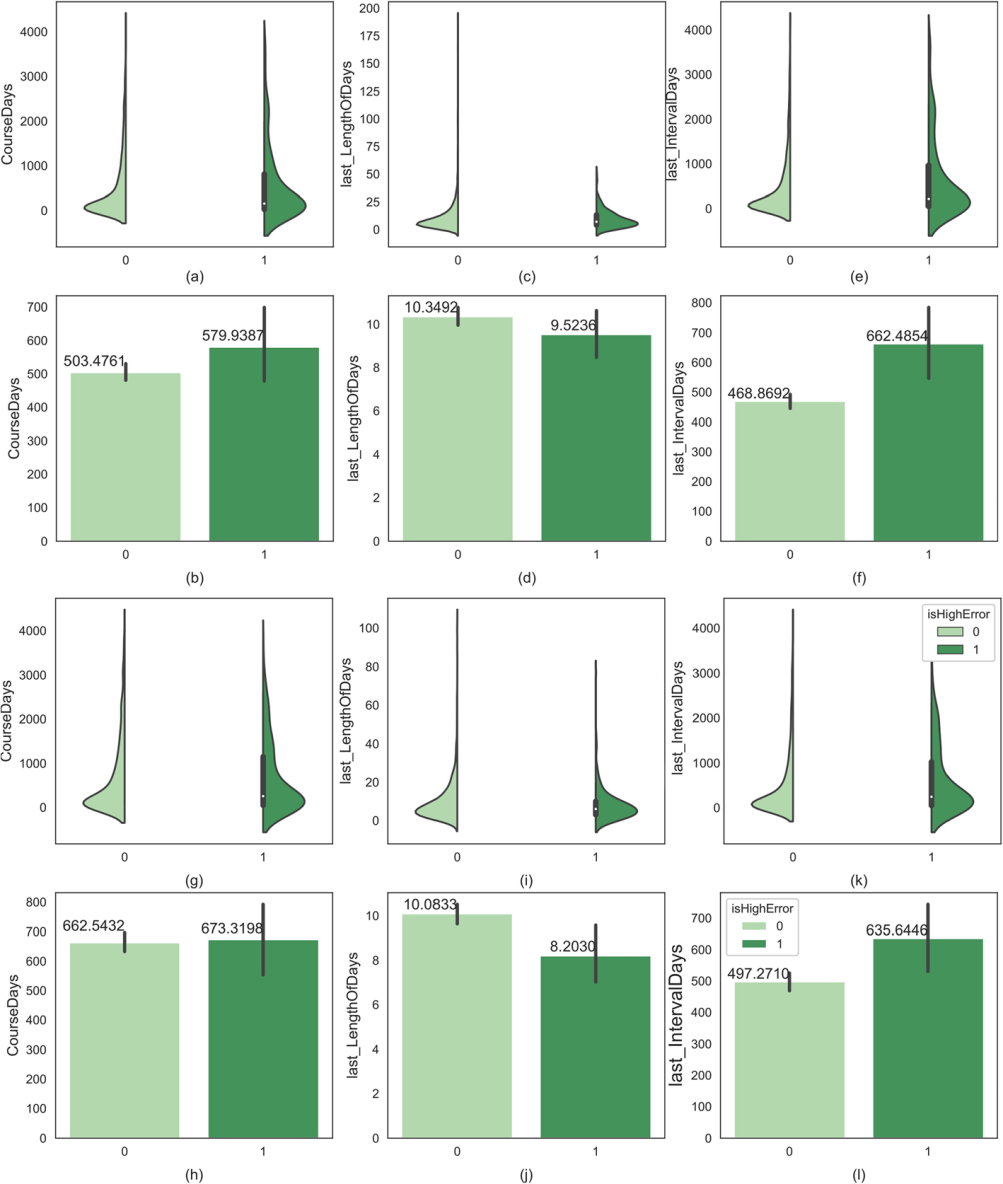
Comparison on the distribution of the hard positive examples and other positive examples in the length of course of disease, the length of last stay in hospital and the interval between the last admission and the last discharge. **a**–**f** Violin diagram and histogram of the three factors in CHF records; **g**–**l** violin diagram and histogram of the three factors in Diabetes records

### Analysis of hard positive examples

In the cross-validation experiments, we found that some positive examples (we pay more attention to the patients with high risk of death) were always misclassified in each model. We called them hard positive examples and collected these hard examples which were misclassified more than half of the total number of times as a test example in cross-validation experiments and found they may have some common characteristics in the distribution of time-dependent factors. Figure 8 shows the comparison of violin diagram and histogram of them with other positive examples with respect to three time-dependent factors, i.e., the length of course of disease, the length of last stay in hospital and the interval between the last admission and the last discharge. From Fig.  8 we have the following observations,

- The average length of disease course of the hard examples is longer than that of the other positive examples. A possible reason is that when the course of disease is prolonged, the development of the disease will be complicated, and other diseases will interfere with the model prediction. For example, compared with non-diabetic patients, the risk of cardiovascular death in patients with diabetes increases with the course of disease.
- The average length of last stay in hospital of the hard examples is shorter. Some hard examples had unforeseen injuries and diseases like burn, contusion, and premature birth, so we speculated that the model could not correctly identify the death risk of patients admitted to hospital due to unforeseen events.
- The interval between the last admission and the last discharge of the hard examples is longer. Essentially, the modified LSTM structure used in DeepMPM may reduce the impact of long-term records. Therefore, when the interval between the last discharge and the last admission is too long, the model may ignore the predictive information in the long-term records, which will lead to the failure to correctly identify the risk of death.

The analysis of hard example can not only help us better understand the possible factors affecting the mortality risk, but also help us design better network structure and improve the prediction accuracy in the future.

## Discussion

We have shown that DeepMPM did work well for mortality risk prediction, and we would try to understand how it works. To know why and how the model makes a prediction can help practitioners to get insight in EHR and understand the model behavior. The framework of DeepMPM offers the potential to provide users with insights into data and model behavior, respectively. In the following, we perform a case study to show the interpretability of DeepMPM.

**Fig. 9:**
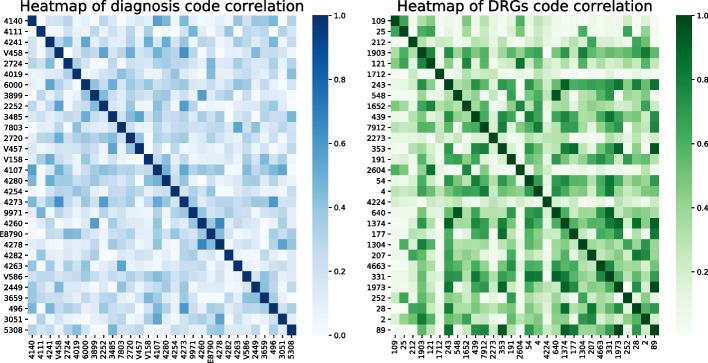
The heatmap of correlation matrix obtained by DeepMPM: **a** pairwise correlation between the diseases; **b** pairwise correlation between DRGs. The deeper the color of the pixel block, the stronger the correlation between the two diseases or DRGs codes represented by rows and columns

### DeepMPM identifies correlation in diseases and DRGs

In DeepMPM, the encoder can provide a global view of the feature correlation in data, by mapping ICD-9 codes and DRGs codes into a high-dimensional space and generating two weight matrices $W_{xemb}\in R^{d\times {|D|}}$ and $W_{pemb}\in R^{d\times {|L|}}$. By calculating the correlation coefficients of the two weight matrices respectively, two correlation matrices $Corr_x\in {R^{{|D|\times |D|}}}$ and $Corr_p\in {R^{{|L|\times |L|}}}$ are obtained, with the element $Corr_{i,j}$representing the correlation coefficient of the codes in row *i* and column *j*. As an example, Fig. 9 displays the heatmap of the correlation matrix of part of diseases and DRGs codes. The darker the color of the pixel block, the closer the relations between the two diseases or DRGs. We check three examples in Fig. 9 to verify its effectiveness.

1. The first row in Fig. 9a corresponds to the correlation between coronary atherosclerosis (“4140”) and other diseases, among which subendo infract (“4107”), generalized heart failure (“4280”), atrial fibrillation and flutter (“4273”) and chronic airway obstruction (“496”) are highly correlated to it. Note that in ICD-9 codes system, the disease codes headed by “4” are all related to circulatory and respiratory diseases, hence the correlation found by the model is reasonable.
2. The fourth row in Fig. 9a shows that, coronary artery disease (“V458”) is highly correlated with diseases such as benign neoplasm of cerebral meninges (“2252”), pure hypercholesterolemia (“2720”) and atrial fibrillation and flutter (“4273”). It is reasonable, since excessive cholesterol content in the blood is an important risk factor for coronary artery disease [51]. Besides, atrial fibrillation and flutter are common arrhythmias in cardiology clinics, which mostly occur in patients with rheumatic heart disease, coronary heart disease, hypertension, etc [52].
3. The first row in Fig. 9b corresponds to the correlation between DRGs of chronic obstructive pulmonary disease(“1403”) and other diseases, and among which the respiratory system signals, symptoms & other diagnostics (“1443”), acute chemical stroke W use of thrombotic agent W MCC (“61”), other respiratory system operating room procedures with complications, comorbidities (“76”), purmonary edema & respiratory failure (“1333”) are highly correlated. It is also reasonable since during respiratory surgery, complications such as pulmonary edema and respiratory failure may occur [53]. Symptoms & other diagnostics (“1443”) is the procedure code used in respiratory surgery, 61 and 76 are related to complications during surgery.

For convenience, we summarize the three examples in Table 6. It can be seen that the correlation matrix can reflect the correlation between diseases or treatments.

**Table 6:** Diseases related to 4140, V458 and 1403 identified by DeepMPM

Example	Related diseases	ICD-9 code
4140	Subendocardial infarction, episode of care unspecified	4107
	Congestive heart failure	4280
	Atrial fibrillation and flutter	4273
	Chronic airway obstruction	496
V458	Benign neoplasm of cerebral meninges	2252
	Pure hypercholesterolemia	2720
	Atrial fibrillation and flutter	4237
1403	Acute chemical stroke W use of thrombotic agent W MCC	61
	Other respiratory system operating room procedures with complications	76
	Purmonary edema and respiratory failure	1333

### The two-level attention reveals relevant visits and diseases to the prediction

**Table 7:** Two-level attention weights of Case 1

Visit ID	ICD-9 code and the disease it represents	Weight
Visit 1 0.2736	1983(Secondary malignant neoplasm of brain and spinal cord)	**0.1619**
	3314(Obstructive hydrocephalus)	0.0403
	1977(Malignant neoplasm of liver, secondary)	**0.1853**
	1970(Secondary malignant neoplasm of lung)	**0.2014**
	1985(Secondary malignant neoplasm of bone and bone marrow)	**0.2337**
	V1006(Personal history of malignant neoplasm of rectosigmoid junction)	**0.1772**
Visit 2 **0.3564**	1983(Secondary malignant neoplasm of brain and spinal cord)	**0.1066**
	431(Intracerebral hemorrhage)	**0.1688**
	78039(Other convulsions)	0.0278
	1970(Secondary malignant neoplasm of lung)	**0.1508**
	1977(Malignant neoplasm of liver, secondary)	**0.1233**
	V452(Presence of cerebrospinal fluid drainage device)	0.0432
	7812(Abnormality of gait)	0.0358
	V1006(Personal history of malignant neoplasm of rectosigmoid junction)	**0.1127**
	4019(Unspecified essential hypertension)	**0.0891**
	V153(Personal history of irradiation, presenting hazards to health)	0.0510
	2518(Other specified disorders of pancreatic internal secretion)	0.0128
	E9320(Adrenal cortical steroids causing adverse effects in therapeutic use)	0.0476
Visit 3 **0.3698**	1977(Malignant neoplasm of liver, secondary)	**0.1282**
	1983(Secondary malignant neoplasm of brain and spinal cord)	**0.0997**
	1970(Secondary malignant neoplasm of lung)	**0.1461**
	5770(Acute pancreatitis)	**0.1503**
	79,902(Hypoxemia)	0.0860
	V1006(Personal history of malignant neoplasm of rectosigmoid junction)	**0.1102**
	V452(Cerebrospinal fluid drainage device)	0.0628
	99591(Sepsis)	**0.0987**
	4019(Unspecified essential hypertension)	0.0823
	25,000(Diabetes mellitus without mention of complication)	0.0354

The visit-level attention weight is displayed under visit ID, while all variable-level attention weights are associated with the ICD-9 codes. Bold values under visit ID indicate that the visit has a relatively higher visit-level attention weight. In the Weight column, bold values indicate that the corresponding ICD-9 code was assigned a relatively higher variable-level attention weight

In addition to revealing the correlations in data, users are also concerned about the mechanism behind each prediction of the model. In DeepMPM, for each prediction of a single patient, the two-level attention LSTM module generate the corresponding weight vectors $\alpha _t$, and $\beta _t$ which reflect the visit-level importance of the records and variable-level importance of features respectively.

We select two patients in the records to illustrate the interpretability of the two-level attention mechanism in DeepMPM, among them one patient (Case 1) eventually died while the other (Case 2) survived. Both cases are correctly predicted by DeepMPM. For both patients, Table 7 and Table 8 show the visit-level and variable-level attention weights for each visit and ICD-9 codes, respectively.

1. From Table 7, we can see that Case 1 was admitted to the hospital for three times, and the main diseases Case 1 suffered were malignant neoplasm of brain, malignant neoplasm of liver, malignant neoplasm of lung and malignant neoplasm of bone. In all the three visits of Case 1, the ICD-9 codes related to malignant tumors are all given high weights by the variable-level attention mechanism. As the condition got worse, more and more serious complications appeared in visit 2 and visit 3, such as intracerebral hemorrhage, unspecified essential hypertension, pancreatic internal secretion, acute pancreatitis and sepsis. Correspondingly, compared with visit 1, the last two visits of Case 1 are given the higher weights by the visit-level attention mechanism.

**Table 8:** Two-level attention weights of Case 2

Visit ID	ICD-9 code and the disease it represents	Weight
Visit 1 **0.4628**	4280(Congestive heart failure, unspecified)	**0.1843**
	4254(Other primary cardiomyopathies)	**0.1598**
	5849(Acute kidney failure, unspecified)	**0.2143**
	2866(Defibrination syndrome)	**0.2071**
	2762(Acidosis)	0.0742
	42,731(Atrial fibrillation)	0.0207
	1749(Malignant neoplasm of breast (female), unspecified)	**0.1412**
Visit 2 **0.3395**	5789(Hemorrhage of gastrointestinal tract, unspecified)	**0.1434**
	4240(Mitral valve disorders)	0.0907
	2851(Acute posthemorrhagic anemia)	**0.1653**
	40,391(Hypertensive chronic kidney disease, chronic kidney disease stage V)	**0.1564**
	4254(Other primary cardiomyopathies)	**0.1921**
	4280(Congestive heart failure, unspecified)	**0.2115**
	4271(Paroxysmal ventricular tachycardia)	0.0186
	56,982(Ulceration of intestine)	0.0081
	53,190(Gastric ulcer, without mention of hemorrhage or perforation)	0.0138
Visit 3 0.1976	71,536(Osteoarthrosis, localized, not specified whether primary or secondary)	0.0898
	4254(Other primary cardiomyopathies)	**0.2391**
	4280(Congestive heart failure, unspecified)	**0.2807**
	4240(Mitral valve disorders)	**0.1897**
	2809(Iron deficiency anemia, unspecified)	0.0245
	V103(Personal history of malignant neoplasm of breast)	**0.1758**

The visit-level attention weight is displayed under visit ID, while all variable-level attention weights are associated with the ICD-9 codes. Bold values under visit ID indicate that the visit has a relatively higher visit-level attention weight. In the Weight column, bold values indicate that the corresponding ICD-9 code was assigned a relatively higher variable-level attention weight
2. As shown in Table 8, Case 2 also has a total of 3 admission records, among which, the first two visits (visit 1 and visit 2) were emergency admissions, and the third one (visit 1) was hospital admission for knee surgery instead of emergency. The main diseases of Case 2 were congestive heart failure, other primary cardiomyopathies, acute kidney failure, malignant neoplasm of breast and mitral valve disorders, etc. These diseases are also given higher weights by the variable-level attention mechanism. Unlike Case 1, Case 2’s condition finally improved. In the last visit, the severe diseases such as acute kidney failure and malignant neoplasm of breast that had occurred before disappeared. Correspondingly, the first two visits of Case 2 were paid more attention by the visit-level attention mechanism.

In summary, for mortality risk prediction, the variable-level attention mechanism accurately captures the main diseases of patients, while the visit-level attention mechanism pays more attention to visits with more serious conditions.

## Conclusions

In this paper, we develop an accurate and clinically interpretable mortality risk prediction model using deep learning. The empirical results show that using disease and treatment information available in EHRs, DeepMPM can achieve more accurate predictions compared with previously reported results. We also show the benefits of using EHRs from patients with multiple diseases and different conditions to predict the mortality risk. The framework of DeepMPM offers the potential to provide users with insights into data correlation and model prediction. Note that DeepMPM is not designed for real-time early prediction of mortality risk since it predicts clinical risks with longitudinal EHRs of patients. In the future work, in order to provides real-time identification of ICU patients at risk, we will develop an early-warning system that integrates multiple monitoring measurements as well as diagnosis and treatment information in EHRs.

## Data Availability

MIMIC III dataset that support the findings of this study are available from PhysioNet but restrictions apply to the availability of these data, which were used under license for the current study, and so are not publicly available. MIMIC III is a restricted-access dataset. To access the files, you must be a credentialed user and sign the data use agreement for the project at https://physionet.org/content/mimiciii/1.4/.

## Abbreviations

ICU: Intensive care unit
EHRs: Electronic health records
SVM: Support vector machine
RNN: Recurrent Neural Networks
CNN: Convolutional Neural Networks
CHF: Congestive heart failure
COPD: Chronic Obstructive Pulmonary Disease
LSTM: Long short-term memory networks
MLP: Multi-layer perceptron
DRGs: Diagnosis related groups
DOD: Date of death
ICD-9: International Statistical Classification of Diseases and Related Health Problems 9th Revision
STLR: Sloped triangular learning rates
TP: True positives
FP: False positives
FN: False negatives
AUC: Area under receiver operating characteristic curve
